# Supplementary material for: The Antiproliferative Activity of High-Dose Somatostatin Analogs in Gastro-Entero-Pancreatic Neuroendocrine Tumors: A Systematic Review and Meta-Analysis
Source: J Clin Med. 2022 Oct 18;11(20):6127. doi: 10.3390/jcm11206127 (PMC9605244; doi:10.3390/jcm11206127)
Supplement: Supplementary file 1 [file jcm-11-06127-s001.zip › jcm-1944996-supplementary.pdf]

Year	Authors	Pan-NENs/all NENs ratio	SI-NENs/all NENs ratio	CR-NENs/ all NENs ratio	G1 rate ^/all NENs ratio	M+ NENs/ all NENs ratio	LD-SSA/all NENs ratio	CHT/all NENs ratio	Primary Tumor resection/all NENs ratio
1994	*Di Bartolomeo et al [24].*	*0.21*	*0.19*	*0.03*	-	*0.65*	*0.79*	*0.12*	*0.39*
1996	*Arnold et al [25].*	*0.33*	*0.42*	*0.04*	-	*0.86*	*0*	*0.20*	*0*
1999	*Faiss et al [26][6]*	*0.10*	*0.30*	*0.17*	-	*1*	*9.33*	*0.10*	*0.53*
2004	*Welin et al [27].*	*0*	*1*	*0*	*0.33*	*1*	*1*	*0*	*0.91*
2009	*Chadha et al [28].*	*0.30*	*0.53*	*0*	-	*0.8*	*1*	*0*	*0.53*
2012	*Ferolla et al [29].*	*0.39*	*0.28*	*0.04*	-	*0.75*	*0.14*	*0.17*	*0.64*
2017	*Strosberg et al [12].*	*0*	*1*	*0*	*0.81*	*0.83*	*1*	-	*0.80*
2018	*Lau et al [30].*	*0.15*	*0.48*	-	*0.06*	*0.66*	*1*	*0*	-
2019	*Lamberti et al [10].*	*0.31*	-	-	*0.53*	*0.60*	*1*	-	*0.64*
2021	*Pavel et al [13].*	*0/1*	*1/0*	*0/0*	*0.56/0.25*	*0.96/0.85*	*1/1*	*0/0*	*0.24/0.46*
2021	*Diamantopoulos et al [11].*	*0.08*	*0.81*	*0.11*	*0.45*	*0.80*	*1*	*0.03*	*0.52*

Supplementary Table S1 – Factors potentially influencing incidence density ratio, disease control rate, and severe adverse events

Legend: Pan-NENs= pancreatic neuroendocrine neoplasms; Si-NENs= small intestinal neuroendocrine neoplasms; CR-NENs= Colo-Rectal neoplasms: SD-SSA= standard dose of Somatostatin analogs; CHT=Chemotherapy**;** ; ^= grade according to 2017 WHO classification; M+= metastatic disease rate
